# EEG-fNIRS self-supervised emotion recognition method based on dual-stream Siamese network

**DOI:** 10.3389/fninf.2026.1894506

**Published:** 2026-08-12

**Authors:** YuGuo Wang, Honglin Wang, Ze Wang, Yan Wu

**Affiliations:** 1 Department of Network Construction and Information Management Center, Jilin Business and Technology College, Changchun, China; 2 School of Computer Science and Technology, Changchun University of Science and Technology, Changchun, China; 3 Department of Computer Science and Technology, Zhongshan Institute of Changchun University of Science and Technology, Zhongshan, China

**Keywords:** EEG, emotion recognition, fNIRS, self-supervised learning, Siamese network

## Abstract

To address the challenges of inconsistent inter-modal contributions, high reliance on labeled data, and limited cross-modal synergy in multimodal emotion recognition, this paper proposes a dual-stream Siamese Self-supervised Emotion Recognition method (DSSER) based on EEG and fNIRS signals. During the pretraining phase, DSSER uses a dual-stream Siamese architecture to extract spatiotemporal features from electroencephalography (EEG) and functional near-infrared spectroscopy (fNIRS) signals. It also aligns their representation spaces through a co-training strategy. This approach eliminates the need for negative samples in conventional contrastive learning. In the fine-tuning stage, a Dynamic Gated Cross-Attention Fusion module is introduced. It assigns modality weights and integrates complementary cross-modal features. Experimental results on a ternary emotion dataset involving 16 participants show that DSSER achieves an accuracy of 98.03% under an intra-subject emotion-recognition protocol. This result reflects performance under the adopted intra-subject setting. It should not be interpreted as subject-independent generalization to unseen participants. The results support the effectiveness of DSSER in improving cross-modal interaction.

## 1 Introduction

Emotion is a complex psychophysiological response to external stimuli and serves as a fundamental component of human cognitive processes. It plays a crucial role in behavioral decision-making and interpersonal communication (Jiang et al., 2023). With the rapid advancement of artificial intelligence, emotion recognition has become an essential technology, enabling machines to perceive human affective states and respond in a more human-like manner. This significantly enhances the naturalness and efficiency of human-computer interaction (Calvo and D'Mello, 2010; Picard, 2001; Sosnowski et al., 2006). Today, emotion recognition holds great promise in various fields, including human-computer interaction, mental health assessment, and intelligent services, and is gradually evolving into a vital bridge between human emotions and intelligent systems (Wang and Zhang, 2024; Ogata and Sugano, 1999; Malfaz and Salichs, 2004).

Traditional emotion recognition methods based on facial expressions, vocal cues, or questionnaires are often affected by subjective bias, cultural differences, and occlusion. When external expressions do not match internal emotional states, the accuracy of these methods may decrease (Yan and Jia, 2023; Li et al., 2022; Adolphs and Anderson, 2018; Li et al., 2023). In contrast, physiological signals can provide a more objective description of emotional states. Electroencephalography (EEG) captures rapid neural activity with high temporal resolution. Functional near-infrared spectroscopy (fNIRS) measures cortical hemodynamic changes with relatively high spatial resolution (Chen J. et al., 2024). Therefore, EEG and fNIRS provide complementary information for emotion recognition. For example, Sun et al. extracted statistical features from EEG and fNIRS. They also used EEG power spectral density (PSD) features from the *θ*, *α*, *β*, and *γ* frequency bands (Sun et al., 2020). Chen G. et al. (2024). constructed graph structures based on statistical and spectral features from EEG and fNIRS. Their method combined graph convolutional networks with capsule attention and achieved an accuracy of 97.91%. Despite these advances, existing fusion methods may not fully consider the changing contributions of different modalities. Li et al. proposed a multimodal physiological signal fusion model. This method uses EEG as the primary modality and treats other signals as auxiliary modalities. It also uses cross-attention to reduce inter-modal inconsistency (Li et al., 2024). However, a fixed primary-modality setting may limit fusion flexibility. The contribution of EEG and fNIRS may vary across samples. To address this issue, this paper introduces a Dynamic Gated Cross-Attention Fusion module. This module estimates the global importance of EEG and fNIRS for each sample. It then uses cross-attention to enhance information interaction between the two modalities. Furthermore, supervised learning methods often rely on large annotated datasets. Such datasets are costly and time-consuming to collect. This limits their practical application in EEG–fNIRS emotion recognition.

Self-Supervised Learning (SSL), as an emerging learning paradigm, aims to learn features by mining the inherent structure of unlabeled data, thereby significantly reducing the reliance on large amounts of labeled data (Jing and Tian, 2019; Wu et al., 2023). Among the numerous self-supervised learning methods, Contrastive Learning is a commonly used technique. Its core idea is to maximize the similarity between samples of the same class while minimizing the similarity between samples of different classes to learn robust feature representations (Dai et al., 2025; Shen et al., 2022). Early contrastive learning methods often drew inspiration from the ideas of SimCLR. For instance, Wen and Yu (2024) constructed positive and negative sample pairs by applying different data augmentations to electroencephalography (EEG) data and used them to train the model. Similarly, Kan et al. (2023) constructed positive and negative sample pairs by determining whether the samples were elicited by the same stimulus, thereby learning semantically consistent feature representations. However, the performance of such methods is highly dependent on the selection of negative samples. Existing negative sampling strategies often introduce sampling bias, which in turn affects the stability of the model and the final recognition performance. To address the aforementioned issues, researchers have proposed a series of improved methods. Grill et al. proposed the BYOL method based on a Siamese Network. This method utilizes an online network and a target network to form a Siamese architecture, training only with two different augmented views of the same source sample as a positive pair. By doing so, it eliminates the dependency on negative samples, thus learning more robust feature representations (Grill et al., 2020). Furthermore, Gao et al. proposed a self-supervised learning method based on a Masked Autoencoder. The core idea of this method is to learn the inherent structure of the data by reconstructing the masked portions, rather than learning features by distinguishing between positive and negative sample pairs (Gao et al., 2024). Although the aforementioned methods have alleviated the dependency on negative samples to some extent, current research on processing multimodal data still commonly adopts a strategy of pre-training each modality separately. This approach of independent unimodal learning overlooks the potential complementary information and intrinsic correlations between electroencephalography (EEG) and functional near-infrared spectroscopy (fNIRS) data, leading to the model's failure to fully leverage the synergistic advantages of multimodal data.

To address these limitations, this paper proposes a dual-stream Siamese Self-supervised Emotion Recognition method (DSSER) based on EEG and fNIRS signals. The proposed framework employs separate branches to extract spatiotemporal features from each modality while aligning their latent feature spaces through a collaborative training strategy. This design enables effective cross-modal feature complementation without relying on negative samples. By preserving modality-specific information through its dual-stream architecture and enhancing semantic consistency via a shared representation space, DSSER effectively resolves the longstanding challenge of balancing modality independence and cross-modal synergy in multimodal self-supervised learning.

## 2 Methods

**Figure 1:**
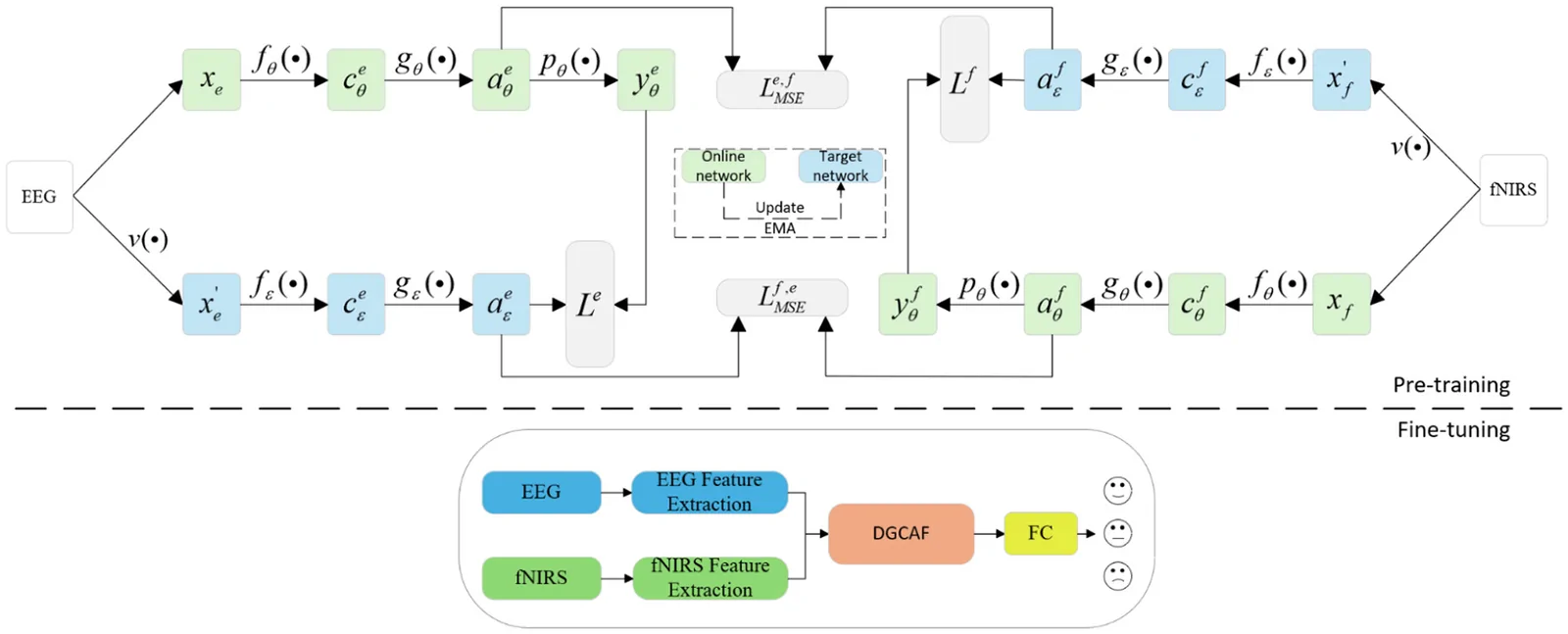
The overall framework of dual-stream Siamese self-supervised emotion recognition (DSSER).

This paper presents the dual-stream Siamese Self-supervised Emotion Recognition (DSSER) framework, a novel approach utilizing electroencephalography (EEG) and functional near-infrared spectroscopy (fNIRS) signals within a dual-stream Siamese network architecture, as depicted in Figure 1. To mitigate the dependency on negative samples, DSSER incorporates a self-supervised learning mechanism that enables collaborative training between the dual streams. This facilitates the joint optimization of spatiotemporal features from EEG and fNIRS, thereby effectively capturing the inter-modal correlation and synergy. The core of DSSER is a dual-stream Siamese network feature extractor. The parameters of the target network, which excludes the predictor module, are updated via an exponential moving average mechanism to enhance training stability. In the fine-tuning phase, a Dynamic Gated Cross-Attention Fusion (DGCAF) module is introduced to achieve complementary fusion of EEG and fNIRS features, thereby augmenting the accuracy of emotion recognition.

### 2.1 Data augmentation

In the model proposed in this article, the input is different views of sample x. Therefore, first of all, it is necessary to enhance the data of sample x. This paper conducted six different data transformations on EEG and fNIRS signals, and compared the effects of different transformations on emotion recognition results through experiments. The following are specific descriptions of these six signal transformations:

(1) Add noise: Add random noise *N*(*t*) with Gaussian distribution to the original signal *X*(*t*).
(2) Scale transformation: Stretching or compressing the amplitude of the original signal. Specifically, the amplitude of the original signal *X*(*t*) is transformed into *α*·*X*(*t*), where *α* is the transformation factor.
(3) Polarity inversion: Polarity inversion changes the sign of the signal amplitude. The transformed signal is defined as −*X*(*t*).
(4) Temporal reversal: Temporal reversal reverses the signal along the time axis. The order of all time points is inverted.
(5) Permutation: Divide the original signal into n non overlapping segments *X*(*t*) = {*x*_1_, *x*_2_, …, *x*_*n*_}, and then shuffle and recombine these segments in time to form a new arrangement signal *X*(*t*) = {*x*_*p*_1__, *x*_*p*_2__, …, *x*_*p*_*n*__}, where {*p*_1_, *p*_2_, …, *p*_*n*_} is a scrambled version of the original order.
(6) Time warp: Divide the original signal into n non overlapping segments *X*(*t*) = {*x*_1_, *x*_2_, …, *x*_*n*_}, randomly select half of the segments, and stretch them using a linear interpolation function *F*(*x*_*i*_, *k*), where *k* is the stretching factor; The remaining fragments are compressed using the function *F*(*x*_*i*_, 1/*k*), where 1/*k* is the compression factor. Finally, connect the time distorted signal segments and adjust them to their original length.

These transformations generate different views for self-supervised learning. Gaussian noise simulates signal interference. Scaling reflects amplitude variation. Permutation and time warping introduce temporal variation. Polarity inversion and temporal reversal are stronger perturbations. They do not have direct physiological interpretations. Future work will explore physiologically constrained augmentation methods.

### 2.2 Dual stream Siamese network

To effectively address the problem of negative sample dependence, this paper designs a dual-stream Siamese network to independently process EEG and fNIRS signals. Each Siamese stream consists of two main branches: an online network and a target network. The online network comprises three components: a feature extractor (encoder), a projector, and a predictor. In contrast, the target network shares a similar architecture but omits the predictor. This asymmetric design has been shown to help prevent model collapse (You et al., 2023). Within this framework, the online network performs self-supervised learning by predicting the target network's representations, thereby enhancing the robustness of feature extraction.

#### 2.2.1 Online network architecture

**Figure 2:**
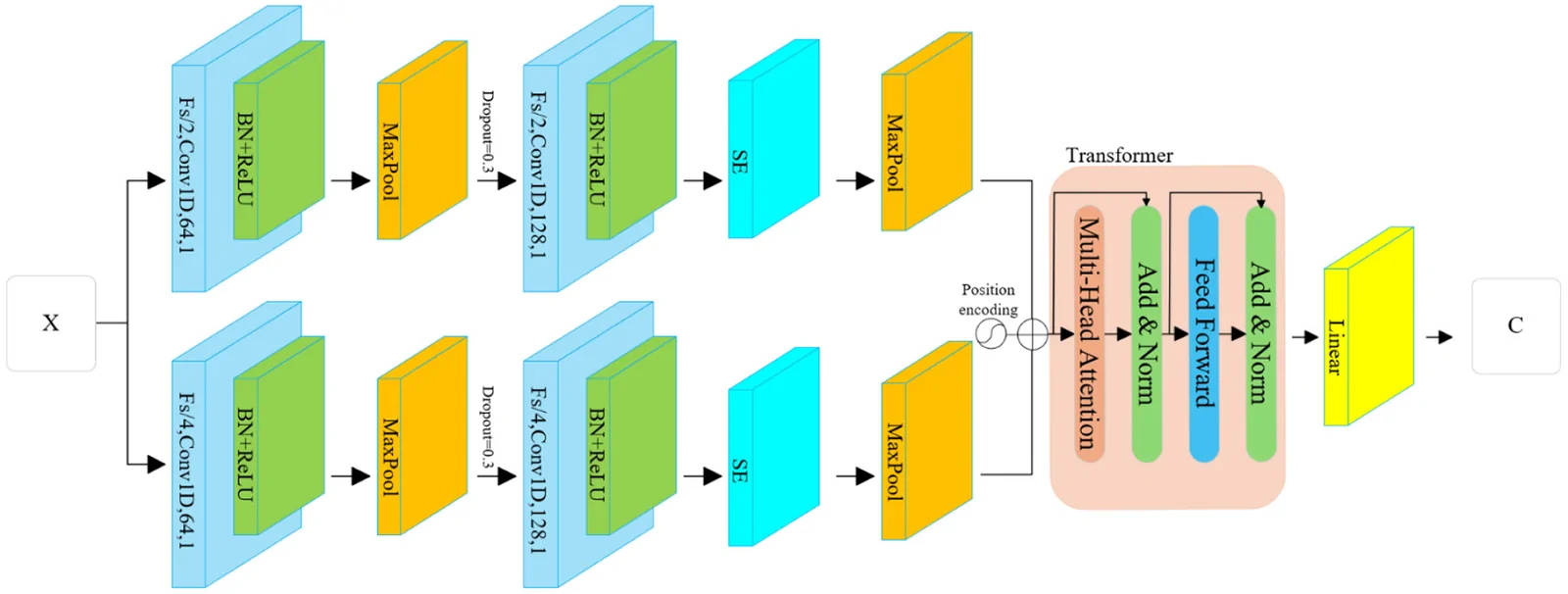
Architecture of the multi-scale CNN-transformer network.

The encoder within the online network is designed to extract spatiotemporal feature representations from both EEG and fNIRS signals. The encoder contains two parallel CNN branches with different temporal kernel sizes. Each branch contains two one-dimensional convolutional layers with 64 and 128 output channels, respectively. The convolution stride is set to 1. Each convolutional layer is followed by batch normalization and a ReLU activation function. Max pooling is used for feature downsampling, and the dropout rate is set to 0.3. An SE module is placed after the second convolutional layer in each branch. The outputs of the two branches are concatenated and then processed by positional encoding and a Transformer encoder. These resultant features are subsequently processed via the projector and predictor modules. The feature extractor is architected in a cascaded fashion, integrating multi-scale Convolutional Neural Networks (O'Shea and Nash, 2015) with a Transformer network (Han et al., 2021). This configuration is specifically intended to capture the time-frequency characteristics and spatial dependencies inherent within the signals. A detailed schematic of this architecture is presented in Figure 2.

To effectively extract time-frequency features from EEG and fNIRS signals, the model employs multi-scale convolutional kernels for feature extraction. The entire convolutional module is composed of several cascaded convolutional blocks. Each convolutional block consists of a convolutional layer, a Batch Normalization layer, and a ReLU activation function. The convolutional operation is responsible for initial feature capture, followed by batch normalization to accelerate model convergence and enhance stability, and then the ReLU activation function introduces non-linear expressive capacity (He et al., 2018). On this basis, a max-pooling layer is connected after each convolutional block to reduce feature dimensionality and retain core information. To further enhance the expressive power of the features, a Squeeze-and-Excitation (SE) module is additionally introduced in the second convolutional block (Hu et al., 2018), aiming to adaptively adjust the weights of each feature channel, thereby strengthening key information and suppressing redundant features. Furthermore, to prevent model overfitting, Dropout technology is applied at the end of each convolutional block (Baldi and Sadowski, 2013). The convolution operation can be mathematically expressed as follows (Equation 1):


$$\begin{array}{l} y_{i}=\overset{k}{\underset{j=1}{\sum }}x_{i-j}\cdot \omega_{j}+b \end{array}$$ (1)

Here, *y*_*i*_ denotes the i-th element of the output sequence, while *x*_*i*−*j*_ represents the (*i-j*)-th element of the input sequence. The term ω_*j*_ corresponds to the j-th weight of the convolution kernel, b is the bias term, and k represents the size of the convolution kernel.

Subsequent to the convolutional operation, the Rectified Linear Unit (ReLU) activation function is applied to introduce non-linearity (Equation 2):


$$\begin{array}{l} \text{ReLU}=\max\left(0,x\right) \end{array}$$ (2)

After extracting preliminary features through multiple convolutional layers, the model first reduces the dimensionality of the feature maps for each modality via a Max Pooling layer (Bieder et al., 2021). This operation effectively reduces the complexity of subsequent computations while preserving the most salient features. Subsequently, the feature vectors output from the two branches are concatenated along the feature dimension to form a unified multimodal feature representation. To enhance the model's generalization ability and prevent overfitting, an L2 regularization strategy is introduced in the subsequent fully connected layers. Before being input into the Transformer encoder, the fused feature vector is first processed by Positional Encoding, which aims to inject positional information about different channels or time steps into the feature vector. On this basis, the Transformer encoder utilizes its core self-attention mechanism to further integrate and extract features, effectively capturing long-range dependencies among them. The calculation process of the self-attention mechanism is as follows (Equation 3):


$$\begin{array}{l} \text{Attention}\left(Q,K,V\right)=\text{softmax}\left(\frac{QK^{T}}{\sqrt{d_{k}}}\right)V \end{array}$$ (3)

Here, *Q*, *K* and *V* denote the query, key, and value matrices, respectively, which are derived from linear transformations of the input sequence *x*. The dimensionality of the key vectors is represented by *d*_*k*_.

Furthermore, the Transformer architecture implements a multi-head self-attention mechanism, wherein multiple self-attention operations are executed in parallel. This methodology enables the model to concurrently attend to information from different representation subspaces, thereby augmenting its capacity to model complex dependencies. The formulation for multi-head attention is expressed as follows (Equations 4, 5):


$$\begin{array}{l} \text{MultiHead}\left(Q,K,V\right)=\text{Concat}\left({\text{head}}_{1},\ldots ,{\text{head}}_{n}\right)\cdot W^{0} \end{array}$$ (4)

$$\begin{array}{l} {\text{head}}_{i}=\text{Attention}\left(QW_{i}^{Q},KW_{i}^{K},VW_{i}^{V}\right) \end{array}$$ (5)

Here, $W_{i}^{Q}$, $W_{i}^{K}$, and $W_{i}^{V}\in {\mathbb{R}}^{d\times d}$ represent the respective projection matrices used to generate the query, key, and value for the *i*-th attention head. The matrix *W*^*O*^ ∈ ℝ^*d*×*d*^ is the output weight matrix that performs a linear transformation on the concatenated outputs of the attention heads. The term head_*n*_ denotes the output of the *n*-th self-attention head.

Subsequently, a feedforward neural network (FFN) is applied to the output of the self-attention mechanism to perform a non-linear transformation and extract deeper feature representations (Equation 6):


$$\begin{array}{l} \text{FFN}\left(Z\right)=\text{ReLU}\left(ZW_{1}+b_{1}\right)W_{2}+b_{2} \end{array}$$ (6)

Here, *W*_1_, and *W*_2_ denote the weight matrices, and *b*_1_ and *b*_2_ represent the corresponding bias vectors for the linear transformation layers. The term ReLU(x) refers to the Rectified Linear Unit activation function. The Transformer model dimension is set to 128, the feedforward dimension is set to 32, and the number of attention heads is set to 2.

Following the feature extraction stage, the projector and predictor modules subsequently process the extracted representations. The projector maps these features into a lower-dimensional latent space, whereas the predictor is tasked with predicting the representation generated by the target network, thereby enhancing the efficacy of the self-supervised learning process. Both the projection and prediction modules are architecturally composed of two fully connected layers, between which batch normalization and a ReLU activation function are applied. The parameters of the online network are iteratively updated through backpropagation, with the output of the target network serving as the regression target.

#### 2.2.2 Target network structure

The architecture of the target network mirrors that of the online network but excludes the predictor module. Its primary role is to provide a stable target representation, facilitating training by reducing noise and instability. The parameters of the target network are updated using an exponential moving average (EMA) of the online network's parameters (Equation 7):


$$\begin{array}{l} \epsilon =\tau \epsilon +\left(1-\tau \right)\theta \end{array}$$ (7)

Here, *θ* denotes the parameters of the online network, *ϵ* denotes the parameters of the target network, and *τ* is the EMA decay rate.

#### 2.2.3 Loss function

In the pre-training phase, this study utilizes encoders with identical architectures and parameter configurations to facilitate the joint training of EEG and fNIRS signals. Specifically, two distinct views are created from the original signals: the original form, x, and an augmented version, x'. These views are subsequently fed into the two branches of the Siamese network, namely the online and target networks, which generate the corresponding output representations *y*_*θ*_ and *a*_*ϵ*_. To assess the similarity between these positive sample pairs, L2 normalization is applied to the outputs, followed by the computation of the mean squared error (MSE) between the prediction and the target projection. This loss formulation incentivizes the online network to accurately predict the representation generated by the target network, thereby improving the quality of the learned features through a self-supervised paradigm as follows (Equations 8, 9):


$$\begin{array}{l} \hat{y_{\theta }}=\frac{y_{\theta }}{{∥y_{\theta }∥}_{2}},\hat{a_{\epsilon }}=\frac{a_{\epsilon }}{{∥a_{\epsilon }∥}_{2}} \end{array}$$ (8)

$$\begin{array}{l} L_{\theta ,\epsilon }={∥\hat{y_{\theta }}-\hat{a_{\epsilon }}∥}_{2}^{2}=2-2\cdot \frac{\langle y_{\theta },a_{\epsilon }\rangle }{{∥y_{\theta }∥}_{2}\cdot {∥a_{\epsilon }∥}_{2}} \end{array}$$ (9)

In addition, to enhance the synergy between EEG and fNIRS features, a normalized modality alignment constraint loss is introduced. Directly minimizing the MSE between unnormalized EEG and fNIRS representations may make the alignment objective sensitive to feature magnitudes. Therefore, before computing the cross-modal alignment loss, the projected representations of both modalities are first L2-normalized, as shown in (Equation 10).


$$\begin{array}{l} {ā}_{\theta }^{f}=\frac{a_{\theta }^{f}}{∥a_{\theta }^{f}{∥}_{2}},\;{ā}_{\epsilon }^{e}=\frac{a_{\epsilon }^{e}}{∥a_{\epsilon }^{e}{∥}_{2}}. \end{array}$$ (10)

The revised cross-modal alignment loss from fNIRS to EEG is defined as follows (Equation 11):


$$\begin{array}{l} L_{\text{align}}^{f,e}=\frac{1}{d}∥{ā}_{\theta }^{f}-\text{sg}\left({ā}_{\epsilon }^{e}\right){∥}_{2}^{2}. \end{array}$$ (11)

Similarly, the normalized representations for the reverse direction are defined as follows (Equation 12):


$$\begin{array}{l} {ā}_{\theta }^{e}=\frac{a_{\theta }^{e}}{∥a_{\theta }^{e}{∥}_{2}},\;{ā}_{\epsilon }^{f}=\frac{a_{\epsilon }^{f}}{∥a_{\epsilon }^{f}{∥}_{2}}. \end{array}$$ (12)

The reverse-direction alignment loss from EEG to fNIRS is defined as follows (Equation 13):


$$\begin{array}{l} L_{\text{align}}^{e,f}=\frac{1}{d}∥{ā}_{\theta }^{e}-\text{sg}\left({ā}_{\epsilon }^{f}\right){∥}_{2}^{2}. \end{array}$$ (13)

Here, *d* denotes the dimensionality of the projected feature vector, and sg(·) denotes the stop-gradient operation. The target representation is used only as a stable regression target and is not updated through backpropagation. After L2 normalization, the alignment loss mainly measures the directional discrepancy between EEG and fNIRS representations and avoids being dominated by feature magnitudes.

Subsequently, *x*′ and *x* are fed into the online network and the target network, respectively, for the computation of the symmetric loss *L*. The symmetric loss function is consequently defined as follows (Equation 14):


$$\begin{array}{l} L=\left(L_{\theta ,\epsilon }+L_{\theta ,\epsilon }^{\prime }\right)/2. \end{array}$$ (14)

Finally, the total loss *L*_total_ is formulated as follows (Equation 15):


$$\begin{array}{l} L_{\text{total}}=L^{e}+L^{f}+\lambda \left(L_{\text{align}}^{f,e}+L_{\text{align}}^{e,f}\right). \end{array}$$ (15)

Here, *L*^*e*^ and *L*^*f*^ denote the intra-modal BYOL losses for EEG and fNIRS, respectively. $L_{\text{align}}^{f,e}$ and $L_{\text{align}}^{e,f}$ denote the normalized bidirectional cross-modal alignment losses. λ is a hyperparameter that balances the contribution of the modality alignment constraint. During each training step, the model is optimized by minimizing the total loss *L*_total_.

#### 2.2.4 Fine tuning

To evaluate the quality of the features learned by the encoder after pre-training, this study fine-tuned the model upon completion of the pre-training phase. First, upon completion of the pre-training phase, the encoder from the target network was retained as the backbone for feature extraction, and its pre-trained weight parameters were loaded. The remaining parts of the model, such as the projection and prediction heads, were discarded. Subsequently, a new feature fusion module and a classification head were connected to the output of this encoder to form a complete model for the downstream classification task. Finally, the classification model was trained in a supervised manner using labeled data. The model was then fine-tuned to better adapt to the specific classification task. Ultimately, the model's classification performance on the downstream task would be used as the core metric to measure the effectiveness of the feature representations learned by the pre-trained model.

Specifically, the feature encoder extracts modality-specific features from EEG and fNIRS signals, which are then fused via an attention mechanism. The fused features are passed to the classification layer for emotion prediction. To enhance the effectiveness of feature fusion, this study introduces a Dynamic Gated Cross-Attention Fusion (DGCAF) module. DGCAF combines a gating mechanism with cross-attention. The gate estimates the global importance of each modality for every sample. It then selects the primary and auxiliary modalities. The calculation process of the gating mechanism is as follows (Equation 16):


$$\begin{array}{l} \begin{array}{c} g_{e}=\text{ReLU}\left(\text{AvgPool}\left(Z_{e}\right)W_{e}\right)\\ g_{f}=\text{ReLU}\left(\text{AvgPool}\left(Z_{f}\right)W_{f}\right)\\ \left[w_{e},w_{f}\right]=\text{softmax}\left(\left[g_{e},g_{f}\right]\right)\\ w_{e}+w_{f}=1 \end{array} \end{array}$$ (16)

Here, $Z_{e},Z_{f}\in {\mathbb{R}}^{c\times d}$ denote the feature representations of EEG and fNIRS, respectively, input into the gating mechanism, where *c* is the number of channels and *d* is the dimension of the feature vector. AvgPool denotes the global average pooling operation, which is employed to extract global feature representations from each modality. $W_{e},W_{f}\in {\mathbb{R}}^{k\times 1}$ are the learnable weight vectors for EEG and fNIRS, respectively. The final weights *w*_*e*_ and *w*_*f*_, computed through the gating mechanism, are used to adaptively determine the primary and auxiliary modalities based on the relative importance of EEG and fNIRS features. The weights are computed from globally pooled features. Therefore, DGCAF performs sample-level modality weighting. It does not model time-resolved modality changes. The weights determine the roles of EEG and fNIRS in cross-attention (Equation 17).


$$\text{primary modalities}=\{\begin{array}{ll} \mathrm{EEG}, & \text{if }w_{e}\geq w_{f}\\ \mathrm{fNIRS}, & \text{otherwise} \end{array}$$ (17)

**Figure 3:**
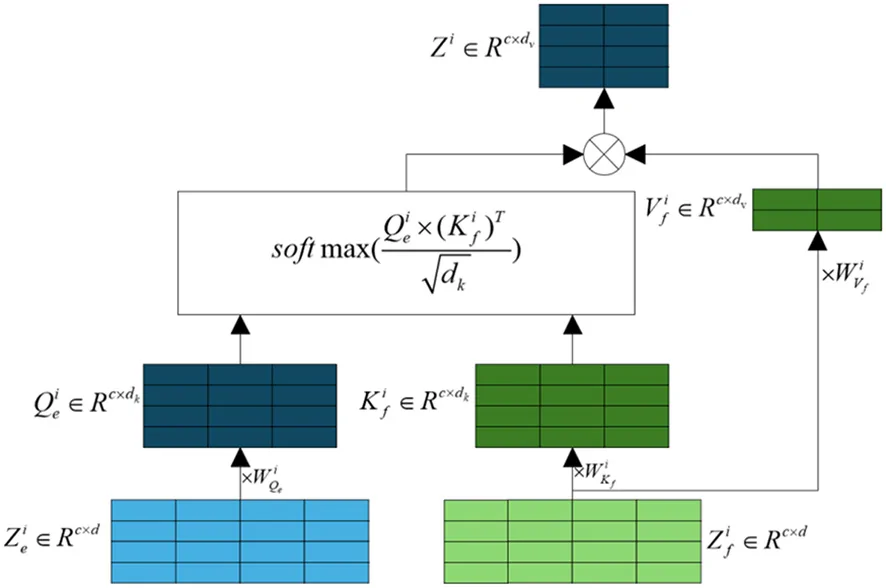
Cross-attention module.


Next, the features of the primary modality are used as queries, while the features of the auxiliary modality serve as keys and values. A Multi-Head Cross-Attention Module (MHCMA) is employed to enhance information exchange between EEG and fNIRS, thereby minimizing representational differences between the two modalities and improving the effectiveness of feature fusion. The MHCMA consists of *I* Cross-Attention Modules (CMAs) operating in parallel. As illustrated in Figure 3, when EEG is designated as the primary modality and fNIRS as the auxiliary modality, each CMA head learns latent features that facilitate cross-modal interaction. The output of the *i*-th CMA head (*i* = 1, 2, …, *I*) is denoted as $Z^{i}\in {\mathbb{R}}^{c\times d_{v}}$, where *c* represents the number of channels and *d*_*v*_ is the dimension of the value vector. The computation is defined as follows (Equation 18):

$$Z^{i}=\mathrm{softmax}(\frac{Q_{e}^{i}\times {\left(K_{f}^{i}\right)}^{T}}{\sqrt{d_{k}}})\;\times \;V_{f}^{i}\;=\mathrm{softmax}(\frac{(Z_{e}^{i}\times W_{Q_{e}}^{i})\times {\left(Z_{f}^{i}\times W_{K_{f}}^{i}\right)}^{T}}{\sqrt{d_{k}}})\times (Z_{f}^{i}\times W_{V_{f}}^{i})$$ (18)

Here, *Z*_*e*_ and *Z*_*f*_ represent the input features from EEG and fNIRS, respectively, fed into the Cross-Attention Module (CMA). *W*_*Q*_*e*__, *W*_*K*_*f*__, and *W*_*V*_*f*__ are the learned weight matrices used to generate the query, key, and value representations for the attention mechanism.

The output of the MHCMA module is represented as follows (Equation 19):


$$\begin{array}{l} \text{Output}=\text{Concat}\left(Z^{1},Z^{2},\ldots ,Z^{I}\right) \end{array}$$ (19)

Following attention-based fusion, the inconsistencies and redundant information between modalities are significantly reduced. Finally, the fused feature representations are passed through the classification layer to perform emotion classification.

## 3 Experiments and results analysis

### 3.1 EEG fNIRS emotion dataset

This study recruited 16 participants (11 males and five females), aged 23–27 years. All participants were right-handed, in good physical health, and reported no history of brain injury or neurological disorders. Prior to the experiment, participants were fully briefed on the experimental procedures. They signed an informed consent form. The study complied with the Declaration of Helsinki and was conducted by the approval of the Ethics Committee of the University Institutional Review Board of Changchun University of Science and Technology (Approval No. CUST-202609-002). During the experiment, they were seated in comfortable chairs facing a computer screen, instructed to focus on viewing the edited movie clips, and to remain as still as possible to ensure data accuracy. The experimental environment was kept quiet and comfortable to facilitate the reliable collection of emotional response data while participants viewed the emotionally evocative movie clips.

**Figure 4:**
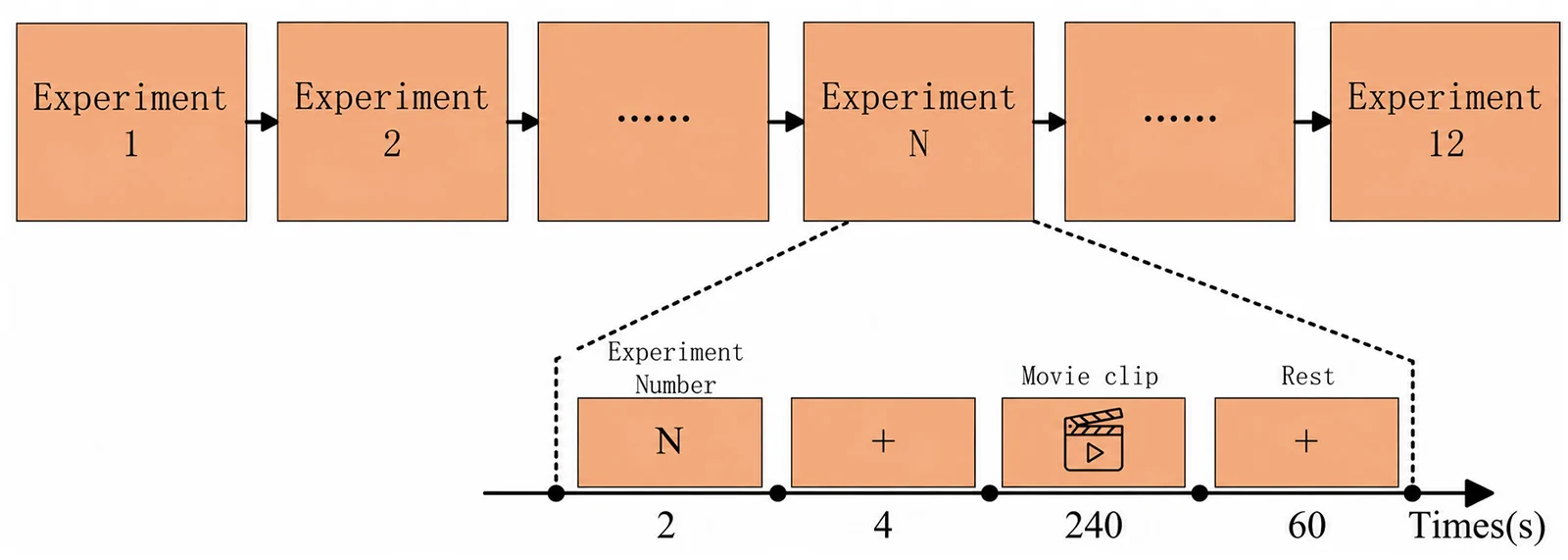
Experimental paradigm.

To elicit different emotional states, 12 movie clips were used as emotional stimuli, sourced from the SEED (Zheng and Lu, 2015; Duan et al., 2013) and SEED-IV (Zheng et al., 2018) datasets as well as selected online video resources. The 12 movie clips were equally distributed across the three emotion categories, including four positive, four neutral, and four negative clips. The clips selected from SEED and SEED-IV had been screened and validated in previous emotion-recognition studies. The additional online clips were selected according to their emotional content and consistency with the three target categories. The experimental procedure was as follows: Participants first sat quietly in a comfortable chair for 30 s, after which a 2-s experiment number was displayed, followed by a 4-s fixation cross (“+”). They then viewed a 4-min video clip, presented in a fixed sequence of positive, neutral, and negative emotions. Following each video, participants rested for 60 s. After viewing each clip, the participants completed the Self-Assessment Manikin questionnaire to report their elicited emotional states. The entire experiment lasted approximately 1 h. The detailed experimental flow is illustrated in Figure 4.

**Figure 5:**
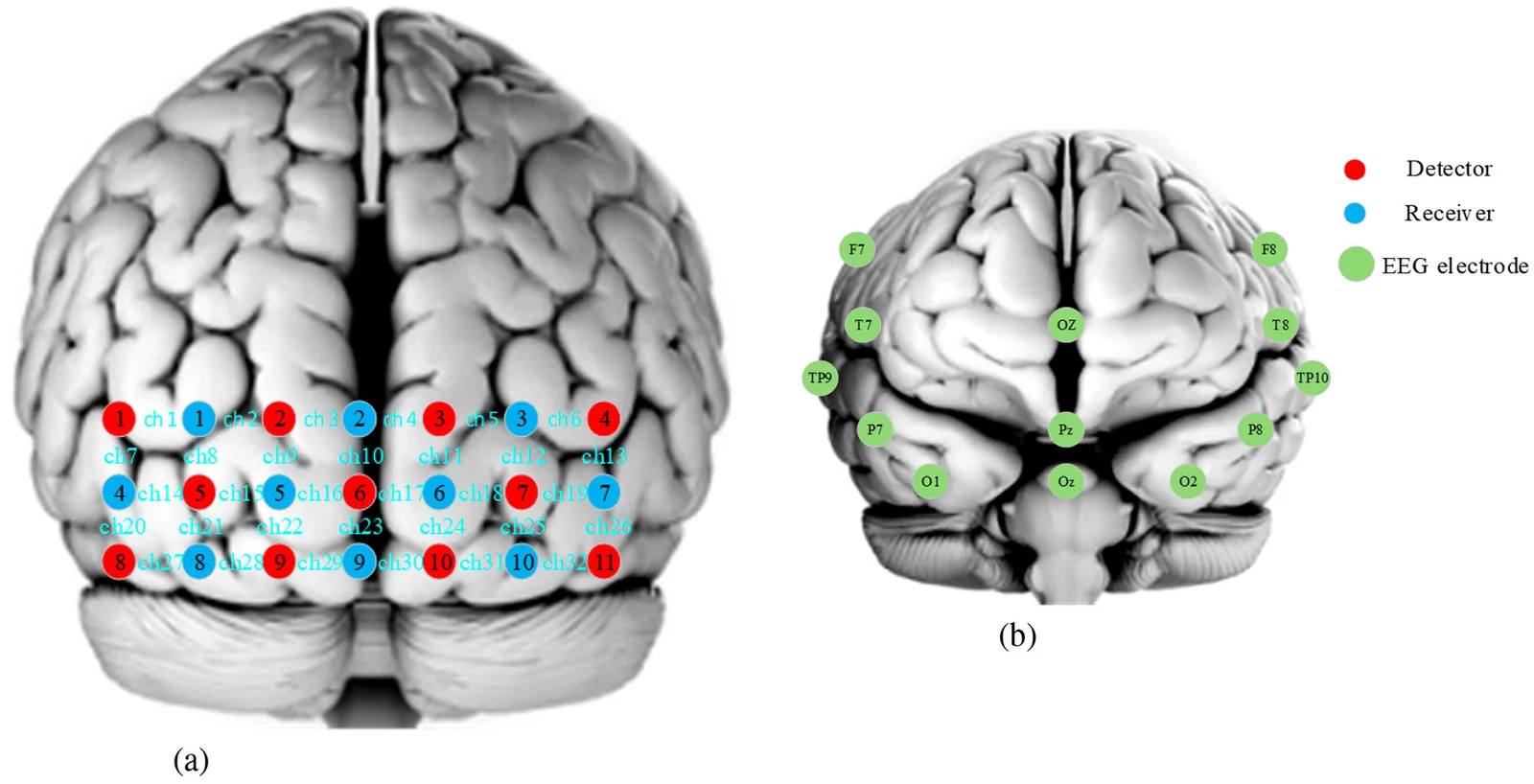
Channel layouts of fNIRS and EEG. **(a)** fNIRS channel layouts. **(b)** EEG channel layouts.

This experiment utilized the LABNIRS system, manufactured by Shimadzu Corporation of Japan (Glaszczka, 2014), to synchronously acquire functional near-infrared spectroscopy (fNIRS) and electroencephalography (EEG) signals. The fNIRS system continuously monitored concentration changes of oxyhemoglobin (HbO), deoxyhemoglobin (HbR), and total hemoglobin (HbT) in the prefrontal cortex by emitting near-infrared light at three different wavelengths (780, 805, and 830 nm). The probe consisted of 11 sources and 10 detectors with a uniform source-detector distance of 3 cm, resulting in a configuration of 32 measurement channels. The sampling frequency of the fNIRS signals was 27.7 Hz. Simultaneously, EEG signals were recorded via 13 electrodes at a sampling frequency of 1,000 Hz. The detailed channel layout for both modalities is illustrated in Figure 5. Only the movie clips from SEED and SEED-IV were reused as emotional stimuli. No physiological recordings or pre-trained models from these public datasets were used. All EEG and fNIRS signals analyzed in this study were newly collected from the 16 participants.

### 3.2 Data preprocessing

This study collected EEG and fNIRS data from 192 video trials (16 participants × 12 videos each), comprising 64 samples each for positive, neutral, and negative emotional states. To enhance data quality and improve emotion recognition performance, both fNIRS and EEG signals underwent preprocessing in MATLAB.

The preprocessing of fNIRS data was primarily conducted using the NIRS-KIT toolbox (Balconi et al., 2015). First, the raw optical intensity signals were converted into optical density signals. Subsequently, the Temporal Derivative Distribution Repair (TDDR) algorithm was employed to automatically identify and correct artifacts caused by head motion. Next, a high-pass and a low-pass filter with cutoff frequencies of 0.01 and 0.5 Hz, respectively, were applied to eliminate physiological noise and high-frequency interference. To further suppress baseline drift, a first-order difference method was also utilized to enhance signal stability. Finally, the sampling rate of the signal was downsampled from 27.7 to 25 Hz. The preprocessing of EEG data was implemented based on the EEGLAB toolbox in MATLAB (Delorme and Makeig, 2004). First, the signals from all channels were re-referenced to the average of the bilateral mastoid electrodes, and their sampling rate was downsampled to 128 Hz. Second, a 0.5–50 Hz Hamming window FIR bandpass filter was applied to preserve the core frequency bands associated with emotional activity. Then, the continuous EEG signals were segmented into independent epochs based on the trigger markers of emotional events. Finally, the Independent Component Analysis (ICA) algorithm was used to automatically identify and remove artifact components generated by activities such as electrooculography (EOG) and electromyography (EMG).

After data preprocessing, the EEG and fNIRS data of 240 s of participants watching videos in each experiment were segmented using a 5-s sliding window non overlapping cutting method, resulting in 9,216 emotion samples. Specifically, each participant completed 12 video trials, and each 240-s trial was segmented into 48 non-overlapping 5-s windows. Therefore, 576 samples were obtained from each participant, resulting in a total of 9,216 EEG–fNIRS paired samples from 16 participants. Each segmented sample retained its corresponding participant ID and trial ID for subsequent data partitioning and analysis.

### 3.3 Experimental setup

This study used a proprietary EEG–fNIRS dataset. The dataset contained 9,216 samples from three emotional states: positive, neutral, and negative. The Transformer module used three main settings. The model dimension was 128. The feedforward dimension was 32. The number of attention heads was 2. During pre-training, the Adam optimizer was used. The learning rate was 1 × 10^−4^. The batch size was 32. The random seed was fixed at 2022. The model was trained for 200 epochs. During model evaluation, the batch size was 64. The learning rate was 1 × 10^−3^. The model was trained for 200 epochs. Model performance was evaluated by five-fold cross-validation. The evaluation used an intra-subject window-level protocol. After segmentation, all 5-s windows were randomly divided into five folds. The data were not divided by trial or by subject. Thus, the reported 98.03% accuracy reflects within-subject window-level performance. It does not represent subject-independent generalization. This window-level split may introduce temporal correlation between training and test samples. Windows from the same participant or the same video trial may appear in different folds. Therefore, the 98.03% result should be interpreted only under this intra-subject protocol. We also report the available cross-subject transfer result. The average accuracy across 16 target subjects was 67.76 ± 14.33%. This result was lower than the intra-subject result. It shows that inter-subject variability remains a major challenge for EEG–fNIRS emotion recognition. The cross-entropy loss function was used as the training objective (Equation 20):


$$\begin{array}{l} \text{Loss}=-\overset{N}{\underset{i=1}{\sum }}y_{i}\log\left({\hat{y}}_{i}\right). \end{array}$$ (20)

Here, *N* represents the number of categories (*N* = 3). *y*_*i*_ denotes the one-hot encoded ground-truth label. ${\hat{y}}_{i}$ denotes the predicted probability distribution generated by the model. All experiments were implemented using PyTorch and conducted on an NVIDIA RTX 4060 GPU.

### 3.4 Experimental results

**Figure 6:**
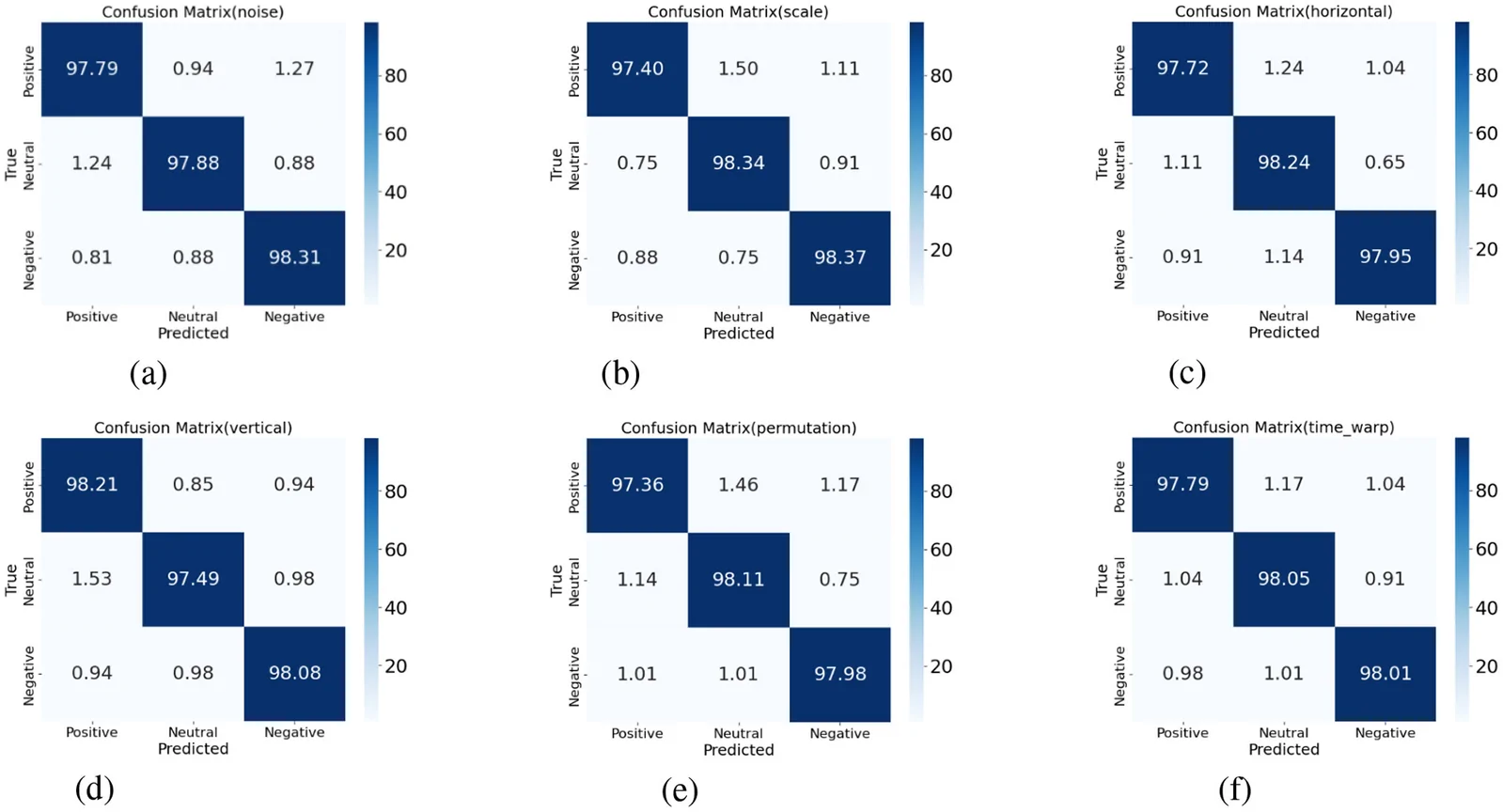
Confusion matrices under different data augmentation strategies. **(a)** Add noise. **(b)** Scale transformation. **(c)** Polarity inversion. **(d)** Temporal reversal. **(e)** Permutation. **(f)** Time warp.

Figure 6 shows the performance confusion matrix of the proposed DSSER model in downstream classification tasks after being pre-trained with different data augmentation strategies based on the fusion of fNIRS (HbO and HbR signals) and EEG data. It can be clearly observed from the figure that regardless of the data augmentation strategy employed. The model achieved relatively consistent recognition results across the six augmentation settings. However, because fold-level variance measures and formal statistical tests were unavailable, these results should be interpreted cautiously: it is not only insensitive to the choice of data augmentation strategy but is also able to effectively learn and extract deep feature representations that are highly correlated with emotional states.

## 4 Discussion and analysis

### 4.1 Comparison with current advanced methods

All baseline methods in Table 1 were re-implemented on our dataset. The same data partition and preprocessing procedure were used. The same training folds, test folds, and evaluation metric were also used. For self-supervised methods, pretraining used only the training data in each fold. The test data were not used during pretraining or model selection. Each method used its original input modality. EEG-only methods used EEG signals. fNIRS-only methods used fNIRS signals. Multimodal methods used paired EEG–fNIRS signals.

**Table 1:** Performance comparison of re-implemented methods under the same evaluation protocol.

Type	Model	Accuracy (%)
Supervised methods	DBJNet (fNIRS) Si et al. (2023)	84.80
	CNN-transformer (fNIRS) Jin et al. (2023)	86.70
	CNN (EEG–fNIRS) O'Shea and Nash (2015)	91.31
	EEGNet (EEG–fNIRS) Lawhern et al. (2018)	90.76
Self-supervised methods	GANSER (EEG) Zhang et al. (2022)	94.53
	ACRNN (EEG) Tao et al. (2021)	94.45
	SGMC (EEG–fNIRS) Chen J. et al. (2024)	95.59
	TFSCL (EEG–fNIRS) Li et al. (2023)	96.84
	MMV-SSTMA-Att (EEG–fNIRS) Chen G. et al. (2024)	94.89
	DSSER (ours)	98.03

Compared with the supervised fNIRS-based models, such as DBJNet and CNN-Transformer, DSSER achieved a higher accuracy under the same evaluation protocol. This result suggests that combining EEG and fNIRS provides more complementary information than using fNIRS alone. DSSER also performed better than CNN and EEGNet, which supports the use of multimodal fusion. Among the self-supervised methods, DSSER achieved the highest accuracy. Compared with EEG-based models, such as GANSER and ACRNN, DSSER benefited from the joint use of EEG and fNIRS. Compared with multimodal methods, such as SGMC, TFSCL, and MMV-SSTMA-Att, DSSER also showed better performance under the adopted protocol. This improvement may be related to two design choices. First, the dual-stream pretraining framework learns modality-specific features and aligns the two representation spaces. Second, DGCAF assigns sample-level modality weights and uses cross-attention for feature interaction. These results support the effectiveness of the proposed framework. However, they should be interpreted within the same dataset, data split, and intra-subject evaluation protocol.

### 4.2 Ablation experiment

To further evaluate the performance of the DSSER model and examine the contribution of each module to the overall effectiveness, this study employed HbOR-EEG data as the input matrix and conducted ablation experiments. Two model variants were designed to validate the effectiveness of the proposed approach:

Variant 1: In the pre-training phase, the EEG and fNIRS feature extractors were pre-trained independently, without collaborative pre-training.

Variant 2: In the fine-tuning phase, the DGCAF module was removed and replaced with a simple cascaded feature fusion strategy.

**Figure 7:**
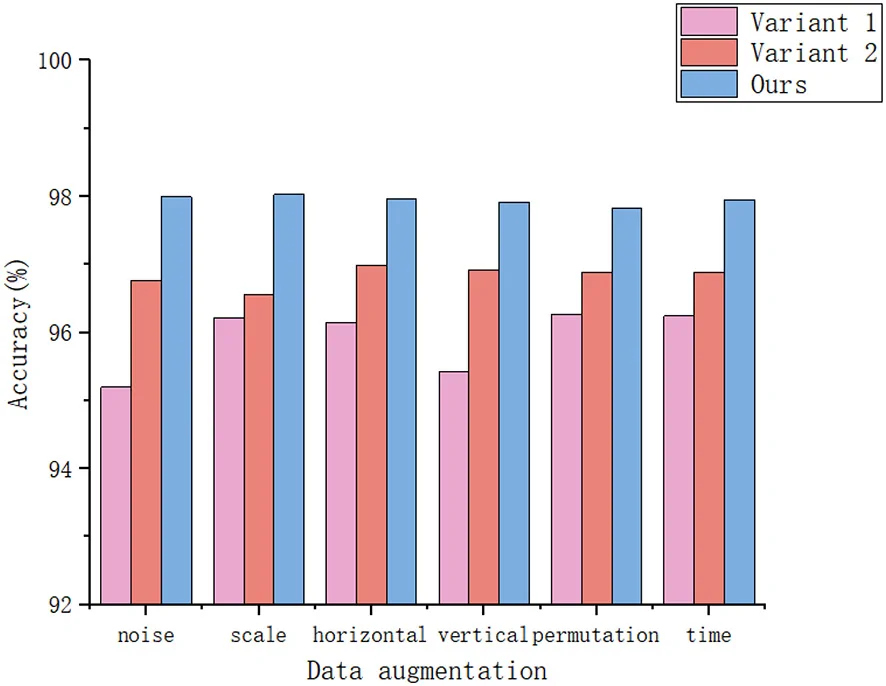
Ablation study results of DSSER.

Figure 7 presents the ablation results. Removing collaborative pretraining reduced the recognition accuracy. Replacing DGCAF with simple feature concatenation also reduced the accuracy. These results support the use of collaborative pretraining and sample-adaptive fusion.

### 4.3 Effectiveness and necessity of combining bimodal data

**Figure 8:**
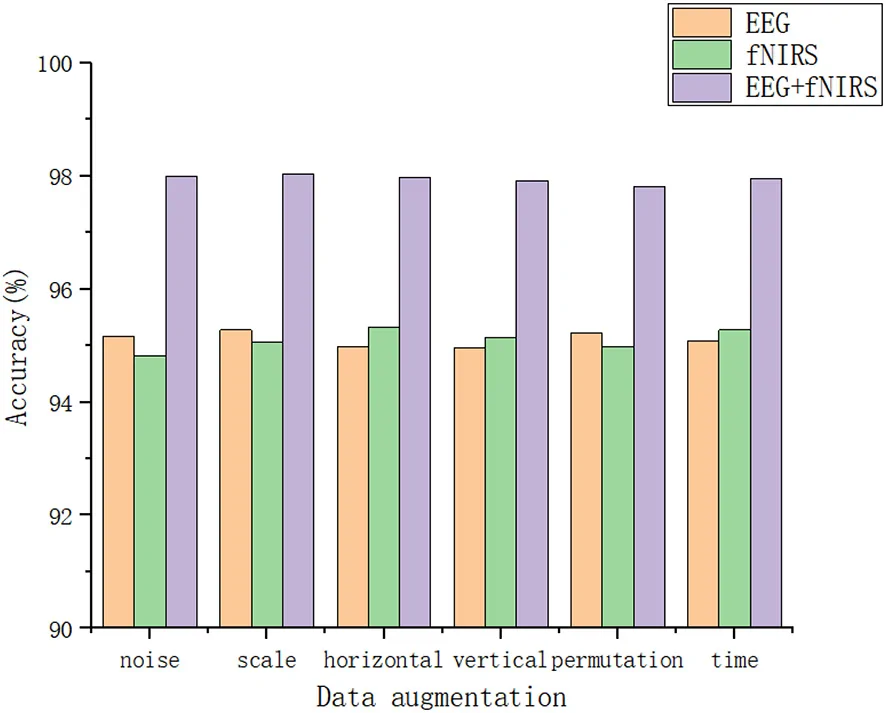
The results of different modes and their combinations.

To validate the advantages of bimodal fusion in emotion recognition, this study compared the performance of unimodal data (EEG or fNIRS) and bimodal data (EEG + fNIRS) across various data augmentation strategies. The fNIRS signals combined the concentrations of oxygenated hemoglobin (HbO) and deoxygenated hemoglobin (HbR) along the channel dimension to preserve comprehensive information on blood oxygenation changes. Figure 8 illustrates the classification accuracies for the three modality configurations (EEG only, fNIRS only, EEG + fNIRS) under six different augmentation methods.

The results demonstrate that bimodal fusion consistently outperforms unimodal approaches in emotion recognition. EEG provides neural information with high temporal resolution. fNIRS provides hemodynamic information with higher spatial resolution. Their fusion achieved higher accuracy than either modality alone. The integration of these modalities achieves complementary spatiotemporal representation, significantly enhancing the model's discriminative capability and robustness to complex emotional patterns. Notably, within the self-supervised pretraining framework, bimodal data facilitates learning of richer latent semantic structures and cross-modal collaborative features, thereby yielding improved classification performance.

### 4.4 Effectiveness of dynamic fusion

To further validate the effectiveness of the main modality determination mechanism introduced in the DSSER model's DGCAF module, this study conducted comparative experiments on emotion recognition performance under different main modality settings, including fixed assignment of EEG or fNIRS as the primary modality, as well as dynamic determination using the DGCAF module.

**Figure 9:**
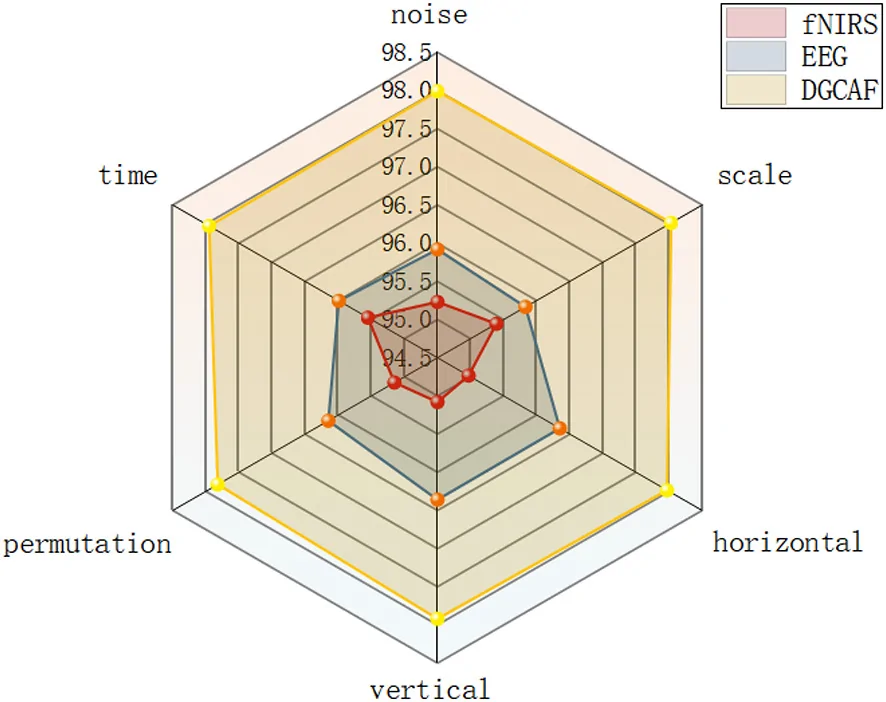
Comparative results of primary modality selection strategies.

Figure 9 presents the comparative results under different primary modality settings. The results demonstrate that the dynamic fusion approach achieves the highest recognition accuracy, indicating variability in the discriminative power of each modality across different emotional states. The dynamic fusion strategy achieved higher recognition accuracy than the fixed-EEG and fixed-fNIRS settings. This result indicates that the relative contributions of EEG and fNIRS may vary across input samples. DGCAF computes sample-level modality weights from globally pooled features and uses these weights to determine the roles of EEG and fNIRS in the subsequent cross-attention operation. However, the current DGCAF module does not explicitly model time-resolved modality changes.

### 4.5 Visualization of classification results

**Figure 10:**
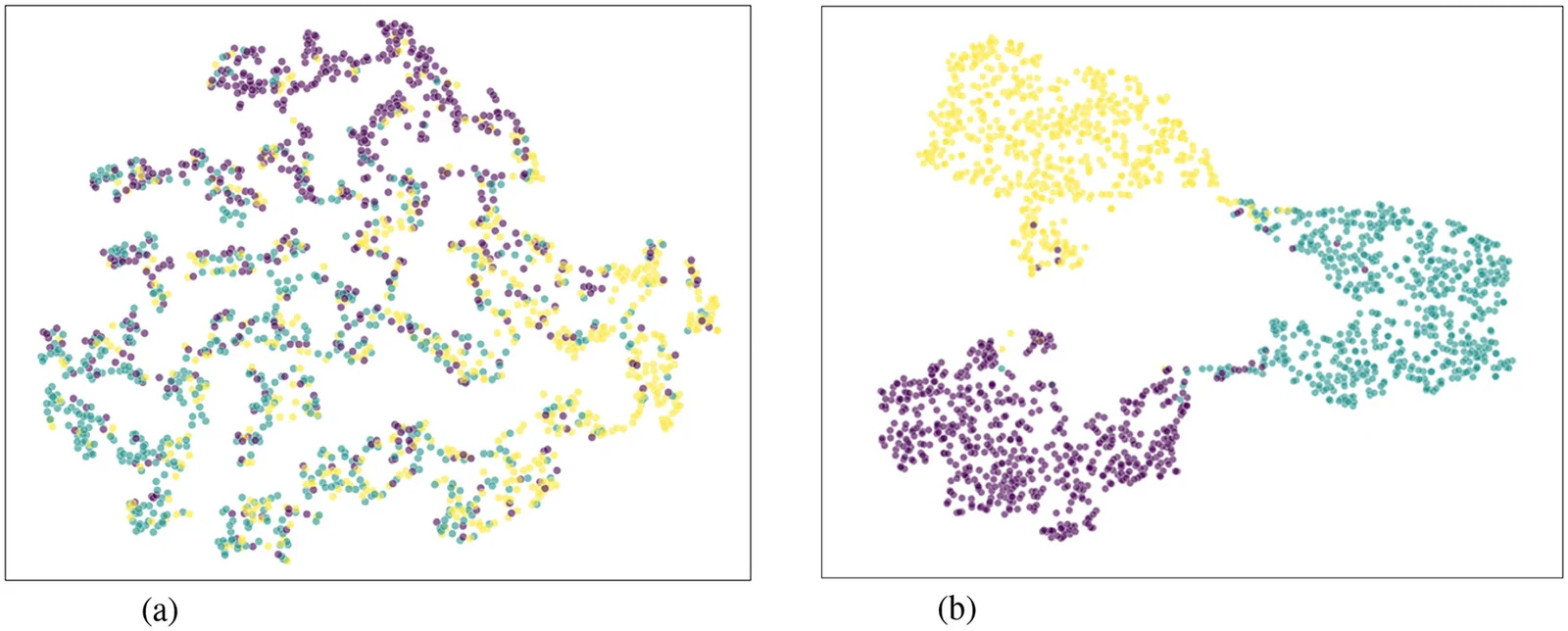
t-SNE plots for 1 and 100% data. **(a)** t-SNE visualization of 1% data. **(b)** t-SNE visualization of 100% data.

To visually assess the DSSER model's performance in downstream classification tasks, t-SNE was employed to project fine-tuned feature embeddings into two dimensions. Figures 10a, b display the dimensionality reduction results obtained using 1 and 100% labeled data, respectively. In Figure 10a, despite the limited labeled data, the model exhibits preliminary clustering tendencies, suggesting initial feature extraction and discrimination capabilities. However, class boundaries remain blurred with notable overlap. In contrast, Figure 10b shows well-defined, compact clusters with clear inter-class boundaries, indicating that sufficient fine-tuning substantially enhances the model's feature representation and discriminative performance.

## 5 Conclusion

This study addresses the critical challenge of signal fusion in multimodal emotion recognition by proposing an innovative framework that integrates electroencephalogram (EEG) signals with functional near-infrared spectroscopy (fNIRS). Using a standardized experimental paradigm, a ternary emotion dataset (positive, neutral, negative) was collected from 16 participants, resulting in a high-quality multimodal dataset for affective computing research. The proposed dual-stream Siamese Self-Supervised Emotion Recognition framework (DSSER) employs a two-stage approach: during pretraining, six data augmentation techniques are applied to generate positive sample pairs, enabling the twin network to learn intra- and cross-modal feature representations in a self-supervised manner without reliance on negative samples. The feature extractor utilizes a cascaded multi-scale CNN and Transformer architecture to effectively integrate temporal and spatial characteristics. In the fine-tuning stage, DGCAF assigns sample-level modality weights. It then uses cross-attention to fuse EEG and fNIRS features. Experimental results demonstrate that DSSER achieves an accuracy of 98.03% under the adopted subject-dependent random window-level evaluation protocol. This result should not be interpreted as subject-independent generalization to unseen participants. In the cross-subject transfer experiment with target-subject fine-tuning, DSSER achieved an average accuracy of 67.76 ± 14.33%. Ablation studies confirm the crucial roles of collaborative pretraining and the DGCAF module, while cross-modal comparisons reveal that EEG–fNIRS bimodal fusion consistently outperforms unimodal approaches across six data augmentation scenarios, underscoring its spatiotemporal complementary advantages and strong application potential. DGCAF does not produce time-resolved modality weights. Future work will explore temporal gating and modality-specific augmentation methods.

## Data Availability

The raw data supporting the conclusions of this article will be made available by the authors, without undue reservation.
